# Catalpol Inhibits Amyloid-β Generation Through Promoting α-Cleavage of APP in Swedish Mutant APP Overexpressed N2a Cells

**DOI:** 10.3389/fnagi.2018.00066

**Published:** 2018-03-19

**Authors:** Zhuo Wang, Xueshi Huang, Pu Zhao, Limei Zhao, Zhan-You Wang

**Affiliations:** ^1^College of Life and Health Sciences, Northeastern University, Shenyang, China; ^2^Shengjing Hospital, China Medical University, Shenyang, China; ^3^Institute of Health Sciences, Key Laboratory of Medical Cell Biology of Ministry of Education, China Medical University, Shenyang, China

**Keywords:** catalpol, amyloid-β (Aβ) peptide, A disintegrin and metalloproteinase domain-containing protein 10 (ADAM10), cAMP-response element binding protein (CREB), Swedish mutant APP overexpressed N2a cell

## Abstract

Amyloid-β (Aβ) peptides play a crucial role in the pathogenesis of Alzheimer’s disease (AD), due to its neurotoxicity. Thus, blocking Aβ generation and aggregation in the brain has been realized as an efficient way for the prevention of AD. The natural product catalpol, isolated from *Rehmannia glutinosa*, has shown neuroprotective activities through inhibiting soluble Aβ production, degrading Aβ peptide, and attenuating Aβ toxicity and neuroinflammatory responses. In the present study, we aimed to evaluate whether catalpol reduce Aβ generation associated with regulating amyloid precursor protein (APP) proteolytic processing. By using Swedish mutant APP overexpressed N2a (SweAPP N2a) cells treated with catalpol, we found that catalpol was not able to reduce the expression levels of β-secretase (BACE-1) and γ-secretase (PS1, APH-1, PEN-2 and Nicastrin). By contrast, catalpol had a significant promotion effect on the expression of α-secretase (ADAM10) and its proteolytic products, sAPPα and C83, suggesting that catalpol reduced the production of Aβ might be involved in non-amyloidogenic APP pathway. In addition, we confirmed that the extracellular signal-related kinase/cAMP-response element binding protein (ERK/CREB) signaling pathways were responsible for the up-regulation of ADAM10 in catalpol-treated SweAPP N2a cells. The present data, for the first time, have demonstrated that the effect of catalpol on the inhibiting Aβ generation might be closely related to α-cleavage of APP processing.

## Introduction

Alzheimer’s disease (AD) is a neurodegenerative disorder mainly occurred in the elderly. The major clinical features of AD include progressive memory deficits and cognitive dysfunction, and the neuropathological hallmarks of AD include senile plaques, neurofibrillary tangles, synaptic dysfunction and neuronal loss (Ali et al., 2015; Ji et al., 2017). The main component of the senile plaque is amyloid-β (Aβ) peptide, which is neurotoxic and may lead to synapse dysfunction, neuronal loss, glial cell activation and oxidative stress (Hardy and Allsop, 1991; Cheng et al., 2006; Eimer and Vassar, 2013; Tu et al., 2014). Therefore, to block Aβ generation and aggregation has been realized as one of the most important strategies for preventing and treating AD.

It is well-known that Aβ is generated from amyloid precursor protein (APP). Through amyloidogenic pathway, the transmembrane APP protein is cleaved by β-secretase (BACE-1) and γ-secretase to generate the small peptide, Aβ (Cao et al., 2016). Conversely, through non-amyloidogenic pathway, APP is cleaved by α-secretase to release soluble sAPPα and C-terminal fragment of 83 amino acids (C83), and hence precludes Aβ production (Zhang et al., 2011). BACE-1, also known as β-site APP cleaving enzyme-1 (BACE-1), is the key enzyme for amyloidogenic APP processing. Inhibition of BACE-1 results in decreasing Aβ production and deposition in AD transgenic mouse brain (Ohno et al., 2007). A disintegrin and metalloproteinase domain-containing protein 10 (ADAM10) is a major α-secretase for APP cleavage, and plays an important role in inhibiting Aβ production (Postina et al., 2004; Kuhn et al., 2010; Yuan et al., 2017). Increasing evidence has suggested that up-regulation of ADAM10 not only reduces Aβ generation, but also inhibits tau hyperphosphorylation and synaptic dysfunctions, and promotes hippocampal neurogenesis (Yuan et al., 2017). It has been generally accepted that inhibition of amyloidogenic APP processing and/or enhancement of non-amyloidogenic pathway is beneficial for inhibiting AD pathogenesis (Kostomoiri et al., 2013). Thus, BACE-1 and ADAM10 have been considered as the major drug targets for combating AD.

Advancements have been made in the use of natural products to prevent AD. Catalpol, one of the iridoid glycoside compounds extracted from *Rehmannia glutinosa*, has shown beneficial effects on neurodegenerative diseases, such as Parkinson’s disease (PD) and AD (Mao et al., 2007; Bi et al., 2008; Huang et al., 2016). Catapol has a wide range of biological activities, includinganti-oxidation, anti-inflammation, anti-aging, anti-tumor and anti-apoptosis properties (Pungitore et al., 2004; Bi et al., 2013; Chen C. et al., 2013; Chen W. et al., 2013; Wei et al., 2014; Yang et al., 2016). Interestingly, catalpol has neuroprotective potential to protect culture cortical neurons against Aβ-induced neuronal apoptosis (Liang et al., 2009). In a cortical neuron-glia culture system, catalpol can attenuate the neurotoxicity induced by Aβ through inhibiting glial inflammation (Jiang et al., 2008a). *In vivo* studies have demonstrated that catalpol protects synaptic proteins from Aβ-induced neuronal damage and improves cognitive abilities in aged rats (Xia et al., 2017). By using D-(+)-galactose intraperitoneal injected mouse as an AD model, catalpol is able to reduce soluble Aβ levels in the brain and relieve learning and memory impairments, through increasing the expression level of insulin degrading enzyme (IDE) and inhibiting oxidative stress (Huang et al., 2016). However, whether catalpol plays a role in mediating the activity of proteolytic enzymes, such as BACE-1 and ADAM 10, has not been fully understood.

In this study, we aimed to evaluate the effect of catalpol on proteolytic processing of APP *in vitro*. By using Swedish mutant APP overexpressed N2a (SweAPP N2a) cells, we found that catalpol could promote the non-amyloidogenic APP processing through up-regulating ADAM10 expression. And the underlying mechanism is related to the extracellular signal-related kinase/cAMP-response element binding protein (ERK/CREB) signaling pathways. The present results indicate that catalpol might be an useful natural product for the treatment of AD.

## Materials and Methods

### Cell Culture and Treatment

The Swedish mutant APP overexpressed N2a (SweAPP N2a) cells were used in the present study (kindly provided by Prof. Huaxi Xu, Fujian Provincial Key Laboratory of Neurodegenerative Disease and Aging Research, Institute of Neuroscience, College of Medicine, Xiamen University). Cells were cultured in Dulbecco’s modified Eagle’s medium (DMEM) with 10% fetal bovine serum (FBS) and L-glutamine in 5% CO_2_ at 37°C. Catalpol (purity >98%) was purchased from Jiangsu Yongjian Pharmaceutical Co., Ltd. (Jiangsu, China). According to different experimental purposes, SweAPP N2a cells were plated in different sized-well plates, and treated with varying concentrations of catalpol and incubation time, as described below.

### Cell Viability Assay

The SweAPP N2a cells were plated in 96-well plates at the density of 3 × 10^4^ cells/mL, and treated with catalpol (0, 200, 400 μM) for 18 h. Control cells were incubated with medium alone. After rinsing with PBS, cells were incubated with MTT (0.5 mg/mL) for 4 h at 37°C. After incubation, the MTT was removed and DMSO (100 μl) was added to dissolve the converted purple dye. Cell viabilities were assessed through measuring the absorbance with a microplate reader at a wave length of 490 nm.

### Aβ ELISA Assay

The SweAPP N2a cells were plated in 96-well plates at the density of 3 × 10^4^ cells/mL and incubated with catalpol (0, 200, 400 μM) for 18 h. After treatment, the culture medium was collected for analyzing the secretory levels of Aβ peptide. The levels of Aβ secretion were measured by using ELISA kits according to the manufacturer’s instructions.

### Western Blot

The SweAPP N2a cells were grown in 60 mm culture dishes at the density of 3 × 10^5^ cells/mL, and treated with catalpol (0, 200, 400 μM). After treatment for 1 h or 18 h, the cells were washed, and then lysed with RIPA lysis buffer [50 mM Tris-HCL (pH 7.5), 150 mM NaCl, 1% NP-40, 1% sodium deoxycholate, 0.1% SDS, 5 mM EDTA, 25 mM NaF, and 2 mM Na_3_VO_4_, 1 mM PMSF] containing 1:100 diluted protease inhibitor cocktail (Sigma, St. Louis, MO, USA). The same amount of protein was separated on a 10% SDS-PAGE and transferred onto PVDF membranes. The membranes were incubated overnight at 4°C with primary antibodies (Table 1). Membranes were washed with TBST and then incubated with HRP-conjugated secondary antibody for 1.5 h. Finally, the immunoreactive bands were detected by Tanon-5500 Chemiluminescent Imaging System (Tanon, China). All the experiments were repeated at least for three times.

**Table 1:** List of antibodies.

Antibody	Host	Dilution	Source (reference)
APP CTF	Rabbit	1:8000	Sigma-Aldrich (A8717)
APH-1	Rabbit	1:2000	Millipore (AB9214)
ADAM10	Rabbit	1:2000	Millipore (AB19026)
Nicastrin	Rabbit	1:1000	Cell signaling technology (#5665S)
PEN-2	Rabbit	1:1000	Cell signaling technology (#8598S)
BACE-1	Rabbit	1:1000	Cell signaling technology (#5606S)
PS1	Rabbit	1:1000	Cell signaling technology (#5643S)
p-ERK	Rabbit	1:1000	Cell signaling technology (#4370S)
ERK	Rabbit	1:1000	Cell signaling technology (#4695S)
p-MEK	Rabbit	1:1000	Sangon (D155091)
MEK	Rabbit	1:1000	Sangon (D161061)
p-PLCG1	Rabbit	1:1000	Sangon (D155046)
PLCG1	Rabbit	1:1000	Sangon (D155196)
p-CREB	Rabbit	1:1000	Cell signaling technology (#9198S)
CREB	Rabbit	1:1000	Cell signaling technology (#9197S)
p-AKT	Rabbit	1:1000	Cell signaling technology (#4060S)
AKT	Rabbit	1:1000	Cell signaling technology (#4685S)
PKA	Rabbit	1:1000	Cell signaling technology (#4782S)
sAPPα	Mouse	1:500	Immuno-Biological Laboratories (11088)
β-actin	Mouse	1:10000	Sigma-Aldrich (A1978)

### Immunofluorescence Staining

The SweAPP N2a cells were cultured on glass coverslips, and treated with 200 μM catalpol for 18 h. After rinsing, cells were fixed with 4% paraformaldehyde for 15 min, and then incubated with 0.1% Triton X-100 for 2 min. The cells were treated with 5% goat serum/PBS for 30 min, and then incubated with ADAM10 antibody (1:500) overnight at 4°C. After rinsing with PBS, cells were incubated with the Alex Fluro 594-conjugated secondary antibody for 1.5 h. The nucleus was stained with DAPI. Images were taken by using a laser scanning confocal microscope (Leica TCS SP8, Germany) with 40× objectives.

### Statistical Analysis

All data were expressed as the mean ± standard error of the mean (SEM) of at least three independent experiments. Differences between means were evaluated with one-way ANOVA for comparison of three or more groups. *p* < 0.05 was considered to be statistically significant.

## Results

### Catalpol Reduced Aβ Generation and Secretion in SweAPP N2a Cells

**Figure 1:**
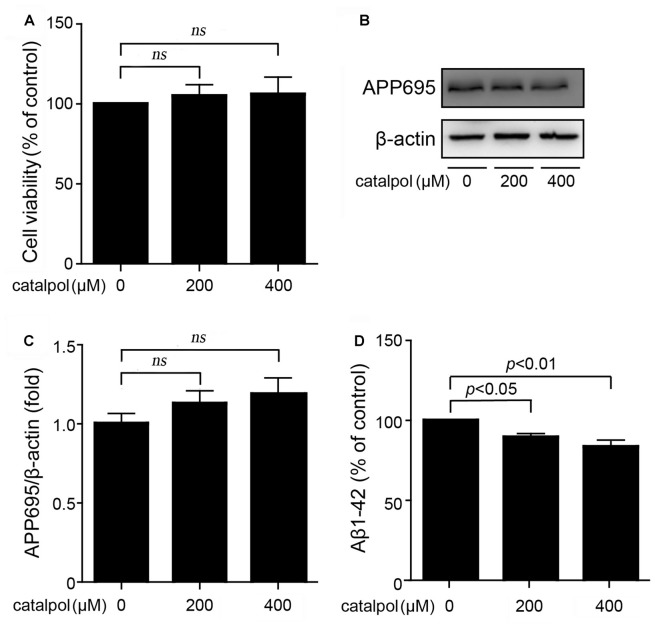
Catalpol inhibited Amyloid-β (Aβ) generation and secretion in Swedish mutant APP overexpressed N2a (SweAPP N2a) cells. **(A)** SweAPP N2a cells were treated with catalpol for 18 h, at the concentrations of 200 and 400 μM, respectively. Cell viability was detected by MTT assay. The results showed that catalpol has no significant effect on cell viability. **(B)** Immunoblot images and **(C)** quantifications showed that catalpol did not affect the expression levels of APP695. **(D)** ELISA analysis showing that catalpol was able to decrease Aβ1–42 levels in the conditional culture medium after treatment for 18 h. The data are expressed as the mean ± standard error of the mean (SEM). *n* = 3. The *p* values were calculated using one-way ANOVA.

It has been reported that catalpol, at the concentration range from 50 μM to 500 μM, has significant neuroprotective activities and no cytotoxic effects on PC12 cells and primary culture neurons (Jiang et al., 2008a,b). However, for SweAPP N2a cells, the protection and cytotoxicity of catalpol has not been evaluated yet. In this study, catalpol at the concentrations of 200 μM and 400 μM were chosen to treat SweAPP N2a cells for 18 h. MTT assay showed that catalpol did not affect the cell viability (Figure 1A).

Through using this culture system, we first analyzed if catalpol treatment could inhibit APP expression and Aβ production. Immunoblotting results showed that the expression levels of APP695 were not affected after catalpol treatment (200 μM and 400 μM) for 18 h in SweAPP N2a cells (Figures 1B,C). Importantly, ELISA detection showed that catalpol treatment markedly reduced Aβ levels in conditional culture medium (Figure 1D). These results suggests that catalpol can inhibit the production and the secretion of Aβ.

### Catalpol Have No Effect on the Expression of β-Secretase and the γ-Secretase Complex

**Figure 2:**
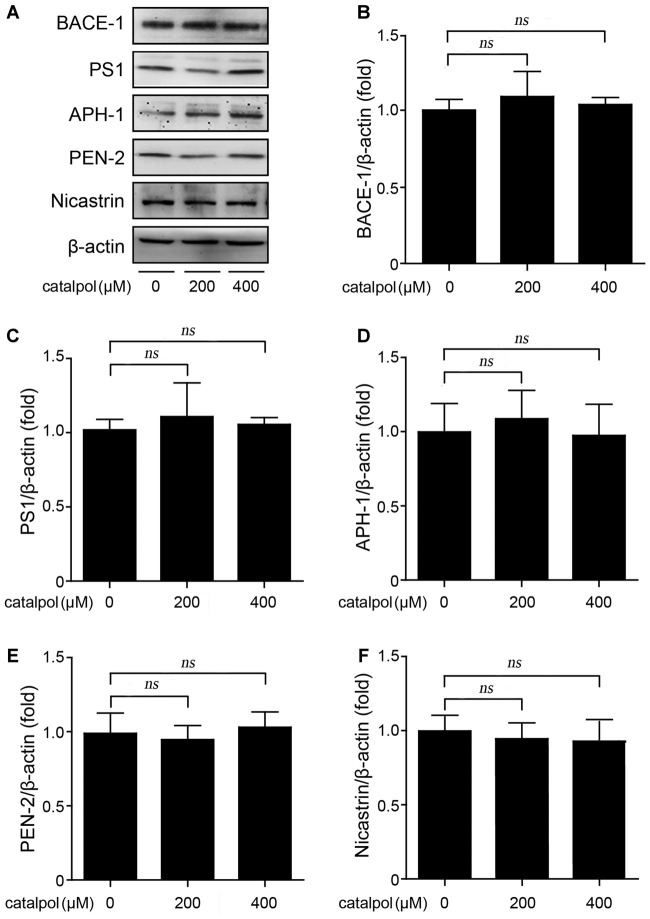
Catalpol did not affect the protein levels of β- and γ-secretase in SweAPP N2a cells. **(A–F)** SweAPP N2a cells were treated with catalpol (200 and 400 μM) for 18 h. Western blot was carried out to detect the expression levels of β-secretase (BACE-1) and the subunits of γ-Secretase (PS1, APH-1, PEN-2 and Nicastrin). **(A)** Immunoblot analysis and **(B–F)** quantifications showed catalpol did not show significant effect on the expression of the detected proteins related to amyloidogenic amyloid precursor protein (APP) proteolytic processing. The data are expressed as the mean ± SEM. *n* = 3. The *p* values were calculated using one-way ANOVA.

We then evaluated whether catalpol inhibited Aβ production is related to amyloidogenic pathway, through detecting the protein levels of BACE-1 (representing BACE-1), and presenilin 1 (PS1), anterior pharynx-defective 1 (APH-1), presenilin enhancer-2 (PEN-2) and Nicastrin, four individual proteins in the γ-secretase complex (Kaether et al., 2006). As shown in Figure 2, western blotting results showed that the expression levels of BACE-1, as well as PS1, APH-1, PEN-2 and Nicastrin, had no significant differences between catalpol treated SweAPP N2a cells and vehicle control cells. These data indicates that catalpol inhibites Aβ generation may be independent of amyloidogenic APP processing in SweAPP N2a cells.

### Catalpol Promoted Non-Amyloidogenic APP Processing Via Enhancing ADAM10 Activity

**Figure 3:**
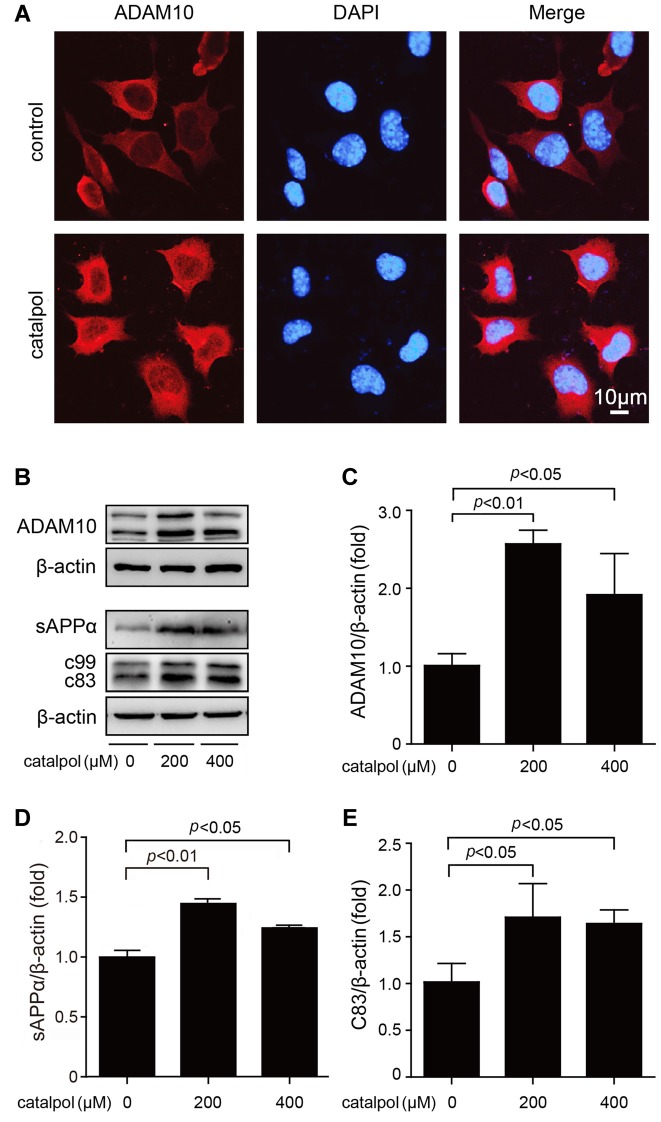
Catalpol increased the expression levels of A disintegrin and metalloproteinase domain-containing protein 10 (ADAM10) and its proteolytic products sAPPα and C83. **(A)** Confocal microscopic images showing an increased immunofluorescence of ADAM10 in SweAPP N2a cells treated with catalpol at 200 μM for 18 h. Nucleus were stained with DAPI. **(B–E)** Immunoblot analysis shows the expression level of ADAM10 (**B**, representative pictures; **C**, quantification), the expression level of sAPPα (**B**, representative pictures; **D**, quantification). The expression levels of C83 (**B**, representative pictures; **E**, quantification) was detected after cells pretreated with the γ-secretase inhibitor L-685, 485. Western blot results showed that catalpol treatments significantly increased the protein levels of ADAM10, sAPPα and C83, after SweAPP N2a cells treated with catalpol (200 and 400 μM) for 18 h. The data are expressed as the mean ± SEM. *n* = 3. The *p* values were calculated using one-way ANOVA.

Next, we analyzed the protein levels of α-secretase (ADAM10) and its proteolytic products, sAPPα and C83, in our culture system. Confocal microscopic analysis showed that catalpol treatment did not affect the distribution of ADAM10. However, the immunofluorescence of ADAM10 in catalpol treated cells was stronger than that in vehicle control cells (Figure 3A). Consistent with the immunefluorescent results, the immunoblotting detection showed that the protein levels of non-amyloidogenic process-related proteins, ADAM10 and sAPPα, were all increased dramatically, after catalpol treatments (200 μM and 400 μM) for 18 h. In addtion, the expression levels of C83 was increased after cells pretreated with the γ-secretase inhibitor L-685, 485 for 1 h (Figures 3B–E). These findings suggest that catalpol-reduced the production of Aβ maybe through promoting α-cleavage of APP.

### Catalpol Promoted ADAM10 Expression Via ERK/CREB Signaling Pathway

**Figure 4:**
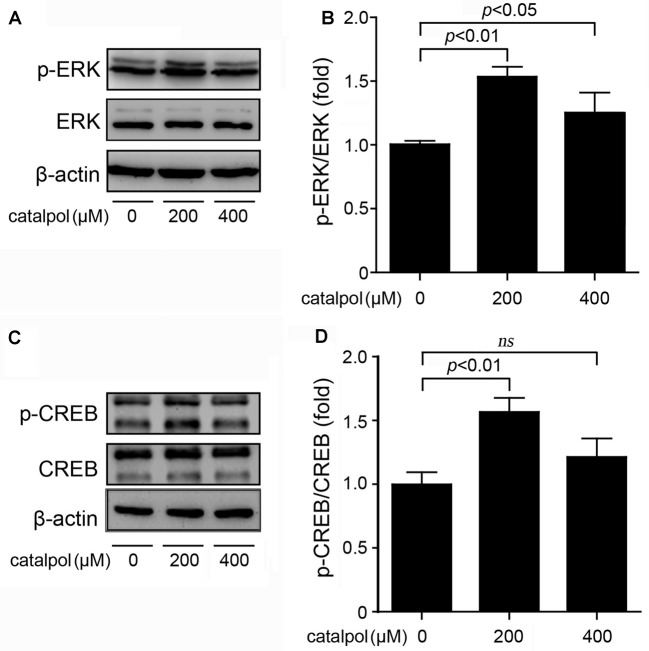
Catalpol activated the extracellular signal-related kinase/cAMP-response element binding protein (ERK/CREB) signal pathway in SweAPP N2a cells. **(A,B)** SweAPP N2a cells were treated with catalpol at 200 μM or 400 μM for 1 h. **(A)** Immunoblotting analyses and **(B)** quantifications showed that the phosphorylation levels of ERK was markedly increased after treatments with catalpol. **(C)** Immunoblotting analyses and **(D)** quantifications showed that catalpol significantly increased the phosphorylation levels of the transcription factor CREB, which is the downstream of ERK. The data are expressed as the mean ± SEM. *n* = 3. The *p* values were calculated using one-way ANOVA.

The expression of ADAM10 is strictly controlled at the transcriptional level (Yuan et al., 2017). As a transcription factor, CREB consensus binding sites are necessary for the transcriptional activity of the ADAM10 promoter (Shukla et al., 2015). Since ERK is the upstream kinase of CREB phosphorylation (Li et al., 2017), the expression levels of ERK and CREB in catalpol-treated SweAPP N2a cells were detected. As shown in Figure 4, immunoblotting results showed that treatment of catalpol at the concentration of 200 μM for 1 h, could significantly increase the phosphorylation levels of ERK and CREB in SweAPP N2a cells.

**Figure 5:**
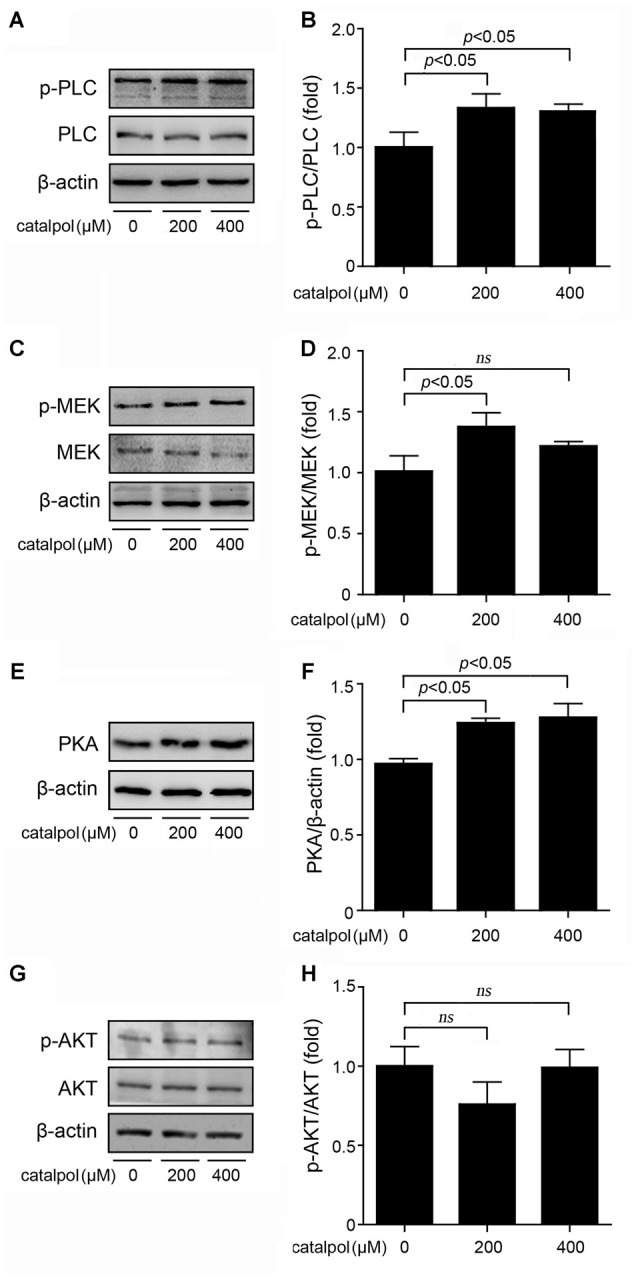
Effects of catalpol on the upstream proteins of CREB signaling pathway. **(A–D)** SweAPP N2a cells were treated with catalpol for 1 h. **(A,C)** Immunoblotting analyses and **(B,D)** quantifications showing the phosphorylation levels of phospholipase C (PLC) and mitogen-activated extracellular signal-regulated kinase (MEK) markedly increased in cells treated with 200 μM catalpol. **(E)** Immunoblotting analyses and **(F)** quantification show that treatments with catalpol at the concentration of 200 μM and 400 μM significantly increased the protein levels of protein kinase A (PKA). **(G)** Immunoblotting analyses and **(H)** quantification showed no significant changes of AKT phosphorylation levels could be detected in catalpol-treated cells compared with vehicle control cells. The data are expressed as the mean ± SEM. *n* = 3. The *p* values were calculated using one-way ANOVA.

The upstream molecules of CREB signaling pathway include phospholipase C/mitogen-activated extracellular signal-regulated kinase (PLC/MEK), cyclic adenosine monophosphate/protein kinase A (cAMP/PKA) and phosphatidylinositol 3 kinase/serine-threonine kinase (PI3K/AKT) signaling. Previous study has shown that PLC/MEK, cAMP/PKA and PI3K/AKT signaling pathways are involved in ADAM10 activation (Fernandez et al., 2010; Shukla et al., 2015). Thus, we detected the protein levels of PKA and AKT following treatment of SweAPP N2a cells with catalpol at the concentrations of 200 μM and 400 μM for 1 h. Our data showed that the phosphorylation levels of PLC and MEK were markedly increased after catalpol treatments (Figures 5A–D). The expression levels of PKA protein were also increased in catalpol-treated cells (Figures 5E,F). However, the phosphorylation levels of AKT had no significantly differences, compared to these from vehicle control cells (Figures 5G,H). These results indicates that PLC/MEK and cAMP/PKA signaling, but not PI3K/AKT signaling pathway, might be involved in catalpol-induced ADAM10 up-regulation, through activated the transcription factor CREB.

**Figure 6:**
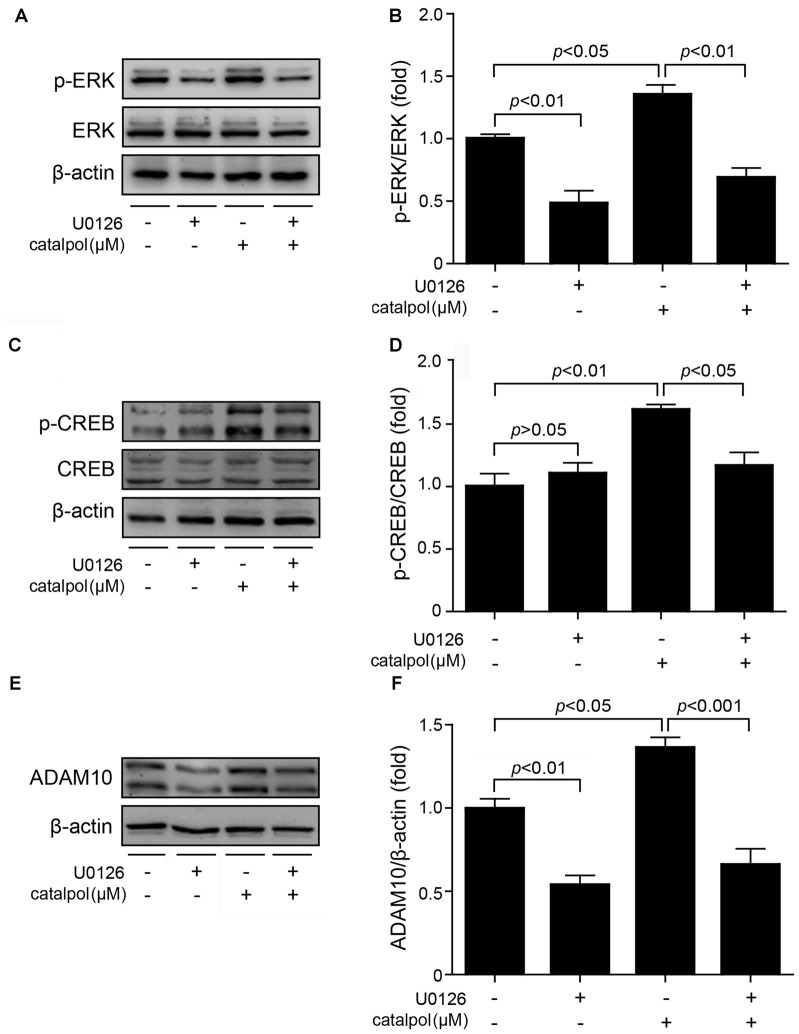
Catalpol promoted ADAM10 expression through ERK/CREB signaling pathway. SweAPP N2a cells were pre-treated with the ERK inhibitor U0126 for 2 h, and then treated with 200 μM catalpol for 1 h. **(A,C)** Immunoblot images and **(B,D)** quantifications show that the expression levels of p-ERK/ERK and p-CREB/CREB were assessed by western blot. The expression levels of p-ERK and p-CREB were markedly decreased in cells treated with both U0126 and catalpol, compared with catalpol-treated cells. **(E,F)** SweAPP N2a cells were pre-treated with U0126 for 2 h, followed by treating with 200 μM catalpol for 18 h. Cells treated with both U0126 and catalpol showed significant decreased levels of ADAM10 protein (**E**, representative pictures; **F**, quantification), compare to these from catalpol-treated cells. The data are expressed as the mean ± SEM. *n* = 3. The *p* values were calculated using one-way ANOVA.

**Figure 7:**
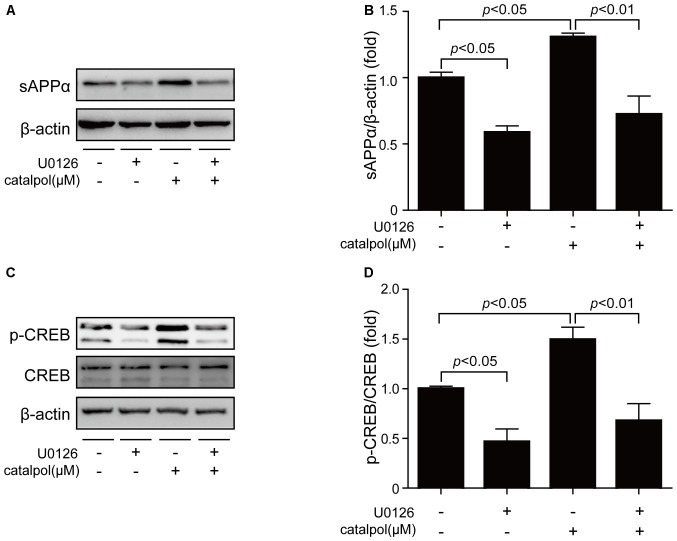
Catalpol promoted sAPPα expression through ERK/CREB signaling pathway. SweAPP N2a cells were pre-treated with the ERK inhibitor U0126 for 2 h, and then treated with 200 μM catalpol for 18 h. **(A)** Immunoblot images and **(B)** quantifications show that the expression levels of sAPPα were assessed by western blot. **(C,D)** The expression levels of p-CREB were markedly decreased in cells treated with both U0126 and catalpol, compared with catalpol-treated cells (**C**, representative pictures; **D**, quantification), compare to these from catalpol-treated cells. The data are expressed as the mean ± SEM. *n* = 3. The *p* values were calculated using one-way ANOVA.

To further confirm catalpol upregulated ADAM10 expression via ERK/CREB signaling, SweAPP N2a cells were pretreated with the ERK inhibitor U0126 for 2 h, and then treated with catalpol (200 μM) for 1 h or 18 h. The phosphorylation levels of ERK and CREB, as well as the expression levels of ADAM10 and sAPPα were measured by Western blot. As expected, U0126 treatment significantly inhibited the expression levels of p-ERK and p-CREB after treated with catalpol for 1 h (Figures 6A–D). Importantly, cells pre-treated with U0126 and then treated with catalpol showed a low level of ADAM10 (Figures 6E,F) and sAPPα (Figures 7A,B), compared with catalpol-treated cells after treated with catalpol for 18 h. The study showed that CREB was a major transcription factor for regulating ADAM10, so we also detected CREB after treated with catalpol for 18 h (Figures 7C,D), and the results were consistent with ADAM10. These suggests that catalpol treatment can lead to increase the expression of ADAM10 via activating ERK /CREB signaling.

## Discussion

The pharmacological functions of catalpol, such as anti-oxidation, anti-inflammation and anti-aging effects, have attracted much attention. The biochemical properties of catalpol, including easy to pass through the blood-brain barrier, and almost no toxic effects, imply that it may serve as a candidate for neuroprotective drug (Wang et al., 2012). Recent studies have indicated that catalpol serves a role in neuroprotection and may be beneficial in preventing AD. It has been shown that catalpol plays a role in protecting primary cultured neurons against Aβ toxicity via inhibiting mitochondrion dysfunction and neuroinflammation (Jiang et al., 2008a; Liang et al., 2009). Catalpol can reduce the oxidative stress, decrease the levels of soluble Aβ, and thus inhibit the plaque formation in a mouse model of AD (Huang et al., 2016). The present study, for the first time, has revealed the underlying mechanism of catalpol on inhibiting Aβ production: catalpol reduces Aβ generation through promoting α-cleavage of APP processing.

It has been generally accepted that blocking the production of Aβ and removing Aβ aggregation will be important strategies for the prevention and treatment of AD. The relative proteolytic efficiency of APP in amyloidogenic pathway and non-amyloidogenic pathway is essential for AD progression due to the production of Aβ generated from the APP proteolytic processing (Gandhi et al., 2004). Hence, targeting the APP proteolytic enzymes, α-, β- and γ-secretase, is able to decrease Aβ generation and deposition in AD brain. In this study, we found that catalpol did not inhibit the expression levels of BACE-1 and γ-secretase (PS1, APH-1, PEN-2 and Nicastrin). These results are consistent with an *in vivo* study showing no effect of catalpol on mediating BACE-1 (Huang et al., 2016), suggesting that Aβ generation reduction induced by catalpol may be not related to amyloidogenic APP processing. We then analyzed whether catalpol inhibits Aβ production is related to non-amyloidogenic APP processing. Our results demonstrated that catalpol could significantly promote the expression levels of ADAM10 and its proteolytic products sAPPα and C83. And the up-regulation of ADAM10 is associated with ERK/CREB signaling pathways. Taken together, this study has revealed that catalpol-inhibited Aβ production is associated with the non-amyloidogenic pathway.

In conclusion, the present study has revealed that catalpol is able to up-regulate ADAM10 expression, promote α-cleavage of APP processing, and reduce Aβ generation. It’s also worth mentioning that catalpol reduce the levels of soluble Aβ40 and Aβ42 in the cerebral cortex and thus inhibit the formation of senile plaques which are regulated by IDE (Huang et al., 2016). As with most of the natural products, the multi-target activity of catalpol on AD prevention needs further study.

## Author Contributions

Z-YW conceived and designed the study. ZW and PZ performed the experiments. Z-YW, XH and LZ participated in data analyses. ZW drafted the manuscript. Z-YW wrote the article. All authors read and approved the manuscript.

## Conflict of Interest Statement

The authors declare that the research was conducted in the absence of any commercial or financial relationships that could be construed as a potential conflict of interest. The reviewer HL and the handling editor declared their shared affiliation.
